# BIM: Enabling Sustainability and Asset Management through Knowledge Management

**DOI:** 10.1155/2013/983721

**Published:** 2013-11-10

**Authors:** Robbert Anton Kivits, Craig Furneaux

**Affiliations:** ^1^Southern Cross University, Lismore, NSW 2480, Australia; ^2^Queensland University of Technology, Brisbane, QLD 4001, Australia

## Abstract

Building Information Modeling (BIM) is the use of virtual building information models to develop building design solutions and design documentation and to analyse construction processes. Recent advances in IT have enabled advanced knowledge management, which in turn facilitates sustainability and improves asset management in the civil construction industry. There are several important qualifiers and some disadvantages of the current suite of technologies. This paper outlines the benefits, enablers, and barriers associated with BIM and makes suggestions about how these issues may be addressed. The paper highlights the advantages of BIM, particularly the increased utility and speed, enhanced fault finding in all construction phases, and enhanced collaborations and visualisation of data. The paper additionally identifies a range of issues concerning the implementation of BIM as follows: IP, liability, risks, and contracts and the authenticity of users. Implementing BIM requires investment in new technology, skills training, and development of new ways of collaboration and Trade Practices concerns. However, when these challenges are overcome, BIM as a new information technology promises a new level of collaborative engineering knowledge management, designed to facilitate sustainability and asset management issues in design, construction, asset management practices, and eventually decommissioning for the civil engineering industry.

## 1. Introduction

Building information management (BIM) is “the use of virtual building information models to develop building design solutions, to design documentation, and to analyse construction processes” [1]. We would suggest that such a definition, while useful, should be extended to include the operational phases of built assets (such as maintenance and decommissioning) and also be applied to the whole area of civil construction. BIM has considerable potential for enhancing the efficiency, sustainability, and effectiveness of civil engineering in all stages of the construction process: planning (or design), construction, facilities management, and decommissioning as it extends the functionality and application of existing computer-aided design (CAD) technologies. The main extension is by linking the 3D built asset model to a relational database that can carry all information related to the built asset [2]. This added functionality provides a mechanism for extended collaborations between designers, engineers, constructors, and facility managers across the life cycle of built assets. Another aspect of BIM is that information, which is created once, can be reused many times, resulting in less errors, greater consistency, clarity, accuracy, and clear responsibility of authorship. This paper argues that BIM holds considerable promise for enhancing a range of activities for civil engineering, with leading authors suggesting 20–30% increase in productivity when using the technology [3]. Despite the prediction that the uptake of BIM in civil construction and facilities management will be slow but inevitable [4], there are some real barriers which need to be addressed in order for this adoption to occur. 

This paper outlines the current promise and future potential for BIM and makes recommendations in relation to how the problems can be addressed. Additionally, while BIM has been primarily explored in relation to buildings, there is little reason why the technologies could not also be applied to other civil infrastructure projects for example, dams, bridges, and tunnels. A set of cases are provided which provide exemplars of how BIM has been implemented. 

From the outset, this paper argues that BIM has the potential for improving all stages of the construction life cycle and has implications for both sustainability and asset management. Accordingly, it is appropriate to firstly provide an overview of the various phases of construction and subsequently how BIM might be implemented in these phases.

### 1.1. Overview of BIM

BIM holds the promise of being an important factor in the built asset industry in the future. It can facilitate the users of all stages of the built asset life-cycle, integrating design, engineering, construction maintenance, and decommissioning information about a built asset project into a single “rich” model. As such, BIM technology enables the use of 3D built asset models to move beyond the design phase and into the construction and maintenance phase of the built asset as well as move the 3D model into a 4D simulation.  Table 1 summarises these implications. 

**Figure 1:**
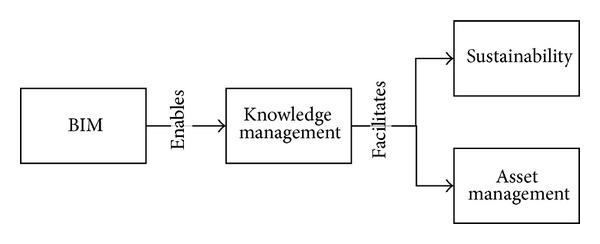
BIM as the foundation to civil engineering sustainability and asset management improvement.

BIM offers the opportunity to develop better cost estimates based on actual elements of the built asset, better design and construction processes and methods, and a means to engage the client in the design phase of the built asset [3].  Figure 1 gives a succinct summary of how BIM can improve sustainability and asset management as it enables collaborative knowledge management across all stages of the asset lifecycle. The enablers such as IT allow for engineering knowledge management by easily sharing information not only within a single organization but also across organizations. This improved and simplified knowledge management in turn facilitates the potential to increase sustainability and asset management for all stages of development.

Much of the potential for BIM has yet to be realised due to the current level of development. As Ashcraft comments, “in current practice, BIM is a hybrid, with several differing approaches being used. Each approach seeks to tighten integration, but the single universal model and perfect interoperability are still aspirations, not achievements” [5]. Ways in which these aspirations can be achieved will be outlined at the end of the paper as future research trajectories. 

## 2. Methods and Methodology

The methods deployed in this paper are an extensive review of the extant academic, policy, and elements of the practitioner literature. A set of extant cases are used as exemplars for showing how BIM has been used in civil engineering projects, with each case purposively chosen to studies discussing the role of BIM in different phases of the construction life cycle. This review goes beyond much of the rhetoric espousing the potential of BIM and carefully considers the barriers as well as benefits of BIM to the civil engineering industry. The cases were deliberately selected in order to learn about the case [6], particularly the application of BIM in various stages of the life cycle, in different sorts of assets, and because the cases generated considerable insight about the phenomenon [7]. Thus, cases were selected in order to provide information on the phenomena of interest [8].

## 3. Application of BIM Case Studies

### 3.1. Applications of BIM in the Past and Present

In the literature, several applications of BIM in the industry can be identified. Table 2 gives an overview of all the identified projects that have implemented BIM, either as a subject of study or as a tool. The most important ones will be discussed in the following section. 

Of this set of cases, four have been chosen as exemplar projects to explore the role of BIM in knowledge management: the Sydney Opera House [9], which is an exemplar of an iconic public asset, and three cases from the United States of America: The Construction Operations Building Information Exchange (COBIE) project [10], US General Services Administration (GSA) [11], and the US Army Corp of Engineers [12]. 

#### 3.1.1. Sydney Opera House

The Sydney Opera House is recognised throughout the world as an iconic symbol of Australia. As FM is considered an ideal tool for the “integration of disparate information management systems, firstly in order to better align FM performance objectives with the organisational objectives, and secondly to further FM objectives in terms of better and more effective FM practices and service delivery” [9]. In a response to this idea, the Sydney Opera House was used to conduct research on FM in practice with the objective of using the research to demonstrate FM as a business enabler and to provide insight into the need to develop a more generic integrated FM solution for the FM industry as a whole. The FM Exemplar Project combines three research streams dealing with Digital Modeling, Services Procurement, and Benchmarking as a whole and then develops collaboration between them. It aims to achieve innovative FM strategies and models that will showcase improved FM performance and promote best practices [9, 13].

The results of the project as a whole and the benefits for using BIM in the FM in general encompass the following benefits as put forward by the project. The key benefit of digital modeling is its accurate geometrical representation of the parts of a built asset in an integrated data environment as follows:

- faster and more effective processes: information is more easily shared and reused;
- controlled whole-of-life costs and environmental data: environmental performance is more predictable and lifecycle costs are understood;
- integration of planning and implementation processes: government, industry, and manufacturers have a common data protocol [9].

#### 3.1.2. COBIE

 “The Construction Operations Built asset Information Exchange (COBIE) is built upon earlier work. COBIE is a built assetSMART initiative of the National Institute of Built Asset Science's Facility Maintenance and Operations Committee, the Facility Information Council and International Alliance for Interoperability, and the National Built asset Information Model Standard. It is a federal government (UA) sponsored effort to support the development of Built asset Information Model(s) (BIM) via information exchange between construction and operations phases” [14]. “The Construction Operations Built asset Information Exchange (COBIE) project, with funding from the U.S. National Aeronautics and Space Administration (NASA), is creating standardized content and format for information handover to operations and maintenance as part of the U.S. National BIM standard (NBIMS). The COBIE approach envisions capturing this information incrementally throughout the facility planning, design, and construction processes” [10].

#### 3.1.3. GSA BIM

In 2003, the U.S. General Services Administration, which is responsible for the management of all civilian federal public built assets in the United States, created its own 3D-4D BIM program, the National BIM Standard. Starting from 2007, the GSA has mandated the use of interoperability and IFC for all major projects [11]. This followed nine pilot projects where BIM was trialed. According to Matta and Kam [15], the direct benefits from these pilot studies include optimizing construction schedule (e.g., reducing the duration by 19%), improving the as-built documentation, uncovering design errors and omissions, and improving the means for communications through 3D-4D BIMs. Furthermore, GSA's initiative has led other federal agencies in the adoption of BIM and has made a major impact on the industry (people, culture, and process), on peer owners, on the attitude towards open standard, and on the importance of establishing an owner's BIM and its requirements.

#### 3.1.4. US Army Corps of Engineers

The US Army has recognised the importance of BIM, and through the US Army Corps of Engineers (US ACE) it is implementing BIM [12]. By 2010, US ACE wants to have 90% compliance with the National BIM Standard (NBIMS) [12]. US ACE actively participates in the development of open standards (NBIMS) for a number of reasons [12] as follows:

- greater competition,
- nonrestrictive contracts,
- government owns the data in long-term format.

What the American Department of Defence (DoD) wants to achieve is the implementation of BIM in a Common Output Level Standard (COLS) that has to provide a common language that is critical for the creation of Joint Base installations. The DoD expects to “significantly … reduce duplication of effort with resulting reduction of overall manpower and facilities requirements capable of generating savings” [16]. The US ACE recognizes that BIM supports their Military Construction (MILCON) program, which covers the construction of facilities and structures as it benefits the design and construction. At the moment, under the Military Transformation program, BIM is a primary deliverable in the US ACE's “FY08 RFP,” the request for a project preproposal. The US ACE expects its design and construction contractors to develop BIM capabilities and their software vendors to use Industry Standards (e.g., NBIMS) and achieve interoperability [12].

At the moment, the four other US federal organisations are effectively implementing BIM; in addition to DoD, these are the U.S. Coast Guard, General Services Administration, NASA, and the Smithsonian Institute.

## 4. Analysis of the Case Studies

By examining those case studies, a set of advantages and disadvantages of BIM in facilitating knowledge management will be explored, followed by the barriers and enablers to full implementation of BIM in civil engineering. The mechanisms of overcoming the barriers and disadvantages will then be discussed. 

BIM is held to offer a range of advantages over hand-drawn or 2D models of built assets. As suggested in the introduction, BIM has emerged alongside a number of other technological and social accomplishments which have enabled BIM as a technology to be developed.  Table 3 provides an overview of how BIM has benefitted the four case studies in the different stages of the asset life cycle. These benefits will be further explored in this section.

### 4.1. Enablers of BIM

For the implementation of BIM, there have been three major enablers. The first is the advent of enhanced IT infrastructure and capability of computers to develop and display 3D models with underlying large databases. The second enabler is the creation of the Industry Foundation Classes (IFC) by the International Alliance for Interoperability (IAI). The third is the increasing worldwide support for BIM. These enablers will be explained in detail below.

The implementation of ICT technology in an organisation poses challenges that need to be overcome. In general, these barriers can be technological in nature, but they can also be related to the need for organisational changes or changes to business processes or even just the speed of implementation [17]. While BIM may contribute a lot of benefits to the construction process, the implementation of this technology presents numerous challengers which need to be overcome. 

#### 4.1.1. Major Advances in Computer Technology and IT Infrastructure

The internet, as a global self-regulated and interconnected network of institutions driven by educational and subsequently commercial priorities, has evolved into an element within a broader “global information society” [18]. The internet is one of the driving forces of globalization, and there is a strong correlation between exporting products and services and internet access at the enterprise level [19]. At a practical level, the internet and roll-out of high speed broadband across OECD countries have facilitated the exchange and sharing of large files across time and space. This has meant that firms can be separated geographically and can operate in separate time zones, and yet the internet enables these firms to collaborate on major projects. Continuous innovations in internet technology and IT infrastructure have in turn increased the performance of BIM. 

Additionally, enhanced computer capacity in processing power and graphics, storage, and memory [20], not to mention better compression algorithms, has meant that larger, more resource intensive files can be created and shared. This enhanced capacity of computers is one of the enablers for BIM technology. The current trend in IT infrastructure, with the latest innovation of fibe optic cables, gives rise to the possibility of sharing even larger data files among users all over the world. BIM is heavily reliant on this infrastructure, since BIM files are large and need to be accessible at all times. Thus, the internet, IT infrastructure, and enhanced capacity of computers have all served as enablers of the creation of large graphical models with huge databases embedded in the models. 

The current development of IT systems can lead to a new organisational architecture and new ways of doing business and delivering services. In the built asset management sector, BIM can enhance the facility management for civil construction. Harris described that, with the need to deliver services differently, the civil construction sector needs to restructure the organisation and engage in dialogue within the organisation and between organisations [21]. This contact and “opening up” of communication channels and developing cooperative arrangements in itself can lead to further synergies in terms of more information sharing, collaboration, and examining new ways to effectively deliver services [21].

The widespread adoption of BIM for civil engineering could possibly be the catalyst to speed up this process, as BIM requires the development of new communication channels and cooperative agreements. The OECD sets out its case for IT in terms of efficiency gains (savings in data collection, information provision, communications with clients and transaction costs) and service improvements (improved customer focus for service delivery and increased accessibility to services) [22].

Enhanced capability is not enough however. There need to be specific protocols which enable the sharing of information between firms and software packages. This is discussed next. 

#### 4.1.2. BuildingSMART (International Alliance for Interoperability)

Interoperability can be described as “the sharing and exchanging of information via integrated technological solutions, no matter what project phase, discipline or participant role in the built asset life cycle” [23]. Although BIM may be considered as an independent concept, in practice, the business benefits of BIM are dependent on the shared utilisation and value-added creation of integrated model data across multiple firms. To access model data therefore requires an information protocol, and although several vendors have their own proprietary database formats, the only open global standard is that published by buildingSMART called the Industry Foundation Classes (IFC).

There are several reasons for the buildingSMART to create a global standard for the built asset sector. One of those is the huge added cost of coordination errors. “Effectively, the historic inefficiencies of the built asset process have driven the industry to look at a new approach to the built asset process. According to the Construction Users Roundtable member companies in the US, it is generally accepted that as much as 30% of the cost of construction is wasted in the field due to coordination errors, wasted materials, labour inefficiencies, and other problems” [24, 25].

In addition to that, in 2004, the National Institute of Standards and Technology (NIST) conducted a study on the cost of inadequate interoperability in the United States' Capital Facilities Industry. The NIST study involved “design, engineering, facilities management, business processes software systems, and redundant paper records management across all facility life cycle phases. It estimated the cost of inadequate interoperability to be roughly $15.8 billion per year, and of these costs, two-thirds are borne by owners and operators” [26].

In order to address this waste of resources, money and time, the IAI is most responsible for promoting interoperability to civil engineering. BuildingSMART is a global, industry-based consortium for civil engineering. It was formed in 1994 and their mission is “to enable interoperability among industry processes of all different professional domains in civil engineering projects by allowing the computer applications used by all project participants to share and exchange project information” [27]. Originally, buildingSMART's main objective was to “define, publish and promote specifications for IFC as a basis for project information sharing in the built asset industry” [28]. BuildingSMART currently has more than 400 members in 24 countries and is the leading interoperability organization [5].

The integration and interoperability of the hundreds of software applications supporting the design and construction of the built environment have been providing one of the most challenging environments for the application of information and communication technologies [29]. BuildingSMART's stimulus in developing the IFC protocol was the recognition that the greatest problem in the construction industry today is the management of information about the built environment. Although every other business sector has embraced IT and adopted industry-specific standards, the construction industry worldwide has stuck to its trade-based roots and dependence on drawings, with a continuing record of poor quality, low investment value, and poor financial rewards [9]. 

The Industry Foundation Classes (IFC) are a set of rules and protocols that describe and store built asset information. According to Batcheler and Howell [30], the “effort to define a single built asset model as one authoritative semantic definition of built asset elements, their properties and inter-relationships” has been largely successful. Bazjanac [31] describes IFC as “the first open object oriented comprehensive data model of built asset that provides rules and protocols for definitions that span the entire life of a built asset.” IFC are also the only such model that is an international standard (ISO/PAS 16739). Presently, IFC are the only nonproprietary intelligent, comprehensive, and universal data model of built assets [31]. 

The creation and existence of these Industry Foundation Classes and the increasingly widespread use of them throughout the industry enable the implementation of BIM in the built asset sector. When all the sectors of the built asset industry are using IFCs as the standard protocol, the sharing of data, as required by BIM, is increasingly easier and barriers due to incompatibility of standards and protocols are reduced. 

Capability of software and hardware to undertake a specific task, to a certain level of performance, is important. The demand for hardware and software that can perform these tasks is what will ensure that there is continued investment in the hardware and software which make this possible. Some government organisations have mandated the use of IFCs, such as Finland and the United States of America. 

#### 4.1.3. Increasing Worldwide Support for BIM

There is an increasing worldwide support for BIM. According to a 2006 survey conducted by the American Institute of Architects (AIA), 16% of AIA member-owned architecture firms have acquired BIM software, and 64% of these firms are using BIM for billable work [1]. The same survey found that “35% of the AIA member-owned firms with an international scope of practice have acquired BIM software, which may be at least partially due to the fact that firms with an international scope tend to be large in terms of staff and billings and may also be working on large projects. But BIM may also simplify overseas projects, as it allows for easy transmission of detailed information quickly over long distances” [1]. As more and more companies start using BIM as the built asset designing and modeling standard, other companies will be forced to follow, to keep a competitive advantage, and to remain interesting as partners for larger firms that require their subcontractors to use BIM as well.

Pragmatically, the number of firms using BIM is quite low, and this may have to do with the adoption cycle of any new technology. Moore [32] provides a useful insight into this by arguing that there is a gap between the early adopters of a new technology and the adoption by the majority of the field. It is in this gap that many innovations fail or falter. Another way of viewing this adoption gap is what Kiviniemi et al. [33, page 56] call the basic dilemma of BIM, which can be described as a paradoxical loop. There is not enough market demand for integrated BIM, because there is not enough measured evidence of benefits of the integrated BIM, because there are no adequate software tools to use integrated BIM in real projects [33].

Some pressure is needed to pull a technology from the promising early start to wide spread adoption by the majority of professionals. Enhancing client demand for the benefits provided by BIM is one catalyst for the adoption of the technology by most of the civil engineering industry. For this to occur, major clients of the civil construction industry would need to be convinced of the benefits of BIM and ensured that all risks had been satisfactorily addressed. 

#### 4.1.4. Summary of Enablers of BIM

In summary, BIM as a suite of technologies has been enabled by the significant improvements in IT infrastructure, the capabilities of computer hardware and software, the increasing adoption of BIM, and the development of IFC which facilitate the sharing of information between firms. 

In current practice, BIM is a hybrid, with several differing approaches being used. Each approach seeks to tighten integration, but the single universal model and perfect interoperability are still aspirations, not achievements [5]. It is likely that the full capability of BIM will not be able to be demonstrated until these barriers and implementations are clearly understood and addressed. 

The civil construction industry, for its part, can enable the adoption of BIM through the use of BIM in various demonstrator projects and supporting the development and adoption of interoperability standards which are necessary precursors to the wider spread utilisation of the technology. 

### 4.2. Promise of BIM

Using a BIM model has a number of advantages over traditional 2D approaches to design and construction. BIM models can enable collaboration between different professionals involved in the design and construction phase of the built asset and can manage changes to the built asset design so that a change to any part of the built asset model is coordinated in all other parts of the model and underlying database, together with the capability of capturing and preserving information for reuse by additional industry-specific applications [5, 34–36]. BIM models can also offer a wealth of information that is generated automatically as the model is created. In turn, this information can be used for cost estimating, project planning and control, and eventually for management of the operation and maintenance of the built asset [37]. The following benefits of BIM have been identified from the literature, which are explored in detail below:

- increased utility and speed,
- enhanced collaborations,
- better data quality,
- visualisation of data,
- enhanced fault finding.

If properly implemented, BIM has clearly some advantages and benefits for civil engineering. However, these advantages are not without some challenges. The technology and business process, upon which BIM is based, does have some disadvantages. Additionally, there are some barriers to be overcome before the full potential of BIM is realised for civil engineering. Just as with benefits and enablers, government policy has a role in the mitigation of the barriers and disadvantages of BIM implementation. 

It is important to note that while BIM is applicable to all stages of construction, Hartman and Fischer [3] note that no single project to date has used BIM in every single phase of construction. Consequently, one of the main hurdles which needs to be overcome is the integration of BIM across all construction phases and by the different participants in a construction project [3, page 3].

Not all benefits are achieved in all phases of the built asset life cycle. Although all benefits are applicable for the design and construction phase, the maintenance and decommissioning phase benefit most from the increased speed and utility, better data quality and the visualisation of the data. In Table 4, these benefits have been summarized.

Having reviewed the benefits of BIM across the project life cycle, it is appropriate to note some of the factors which are enabling the growth and uptake of the technology.

While some governments have mandated the adoption of BIM, this has been following extensive use of pilot studies which have trialled a number of BIM applications in a number of settings (e.g., GSA in the USA [38]). Demonstration projects are likely to be necessary prior to the use of other policy instruments such as education, regulation, and policy. 

### 4.3. Problems with BIM

Just as there are a number of advantages to the use of BIM in civil engineering, there are a number of challenges. Those discussed here are the single model, interoperability, added work for the designer, and the sheer size and complexity of BIM:

- a single detailed model,
- interoperability,
- added work for the designer,
- the size and complexity of BIM,
- Trade Practices implications.

These disadvantages are mostly identified in the design and construction phase of the built asset life cycle. These disadvantages mainly have to do with the differences in which architects and engineers work. Although all benefits are applicable for the design and construction phase, the maintenance and decommissioning phase benefits most from the increased speed and utility, better data quality, and the visualisation of the data. In Table 5, these benefits have been summarized.

In the very traditional profession of civil engineering, new technologies are not easily introduced. In general, when a new technology is introduced, there is a certain period of time in which the claims about the potential of the technology need to be examined, tested, and verified. Particularly, the AEC/FM industry is known for the very long adoption periods of promising technologies, despite the highly mobile workforce that must collaborate with a range of on- and off-site personnel and make use of the large volumes of information [37]. Even while new standards are being, and have been, developed (IFCs), the adoption of these standards has been slow. Due to this slow speed of adoption, it is very difficult to demonstrate the benefits of these standards [25]. 

The first barrier is addressing the legal issues involved with BIM and the interorganisational way of operating, using one single complex project file. In relation to BIM, the legal concerns identified to date include risk allocation, standard of care, privacy and third party reliance, economic loss doctrine, who is the designer, and intellectual property [5].

These concerns are grouped together and in this section the IP, liability and risk, and contractual issues are treated.

The foregoing section has dealt with several barriers of implementation for BIM. To give a short overview of all the barriers, Table 6 gives a summary of these barriers.

Not only the technical limitations of BIM have been identified but also have the legal, social, and financial barriers that can prevent a successful implementation of BIM. For the further development of BIM, new business models will have to be designed that assist the integration of BIM as a project delivery method, rather than the old methods where it is attempted to integrate these new technologies into conventional practices [5]. “This rethinking must necessarily include a disavowal of the build it and they will use it' mentality that infiltrates much of web-enabled thinking” [39]. Given the potential of BIM as a set of technologies, it is certainly time to address the numerous financial, intellectual, legal, and organisational issues which currently inhibit the widespread adoption of BIM. As has been outlined above, it is likely that a range of policy instruments would be required to address all of these concerns policy and regulation, financial support allowances and incentives, education, and the trialing of the technology in numerous settings. 

### 4.4. Longer Term Potential of BIM

#### 4.4.1. The Utility of BIM in the Design Phase

Historically, designing a built asset involved drawing a two-dimensional (2D) image of the built asset on paper and making hard copies for other participants to use in the next phase construction. In the early 1980s, architects started using CAD, or computer-aided design, which allowed designs to be created on a computer in a 2D format and copied more easily. In the evolution from paper-based drafting to 2D CAD, the relationship between designers and contractors remained stable, with little change noted in procedures [34–36]. The reason for this is that while CAD improved processes for architects as they designed built assets, the end product—a 2D drawing—was effectively the same. CAD systems produced marginal benefits for many organisations over conventional drawing methods. This was because the electronic design invariably became committed to a hard-copy version at numerous stages. The electronic version was dispensed with and at each stage the drawing had to be recreated from scratch [40]. 

While CAD enabled drawings to be created on a computer, in the end, the drawings were converted to 2D hard copy and handed over to the contractor. So up until the early 1990s, innovations driven by ICT only affected the design stage of the construction process. The remainder of the construction process remained relatively unchanged. 

The introduction of Object Oriented CAD (OOCAD) systems in the early 1990s involved the replacement of 2D symbols in CAD drawings with built asset elements (objects) which were capable of representing the behavior of common built asset elements. The key improvement of this technology was that these built asset elements could have nongraphic attributes assigned to them and associations between the various elements of a built asset to be made [30]. Additionally, this new 3D computer technology enabled designers to better visualise a built asset, by being able to rotate the built asset and view it from multiple angles. The third parallel development in the 1990s was the increasing use of internet for digital data sharing [41, 42]. This increased use of object oriented modeling in the design phase and the capability of the Internet to enable information sharing between geographically and temporally distant firms resulted in the emergence of BIM as a set of technologies. In line with the increasing computer hardware and software capability, most CAD vendors have launched more powerful object-based CAD software in recent years. These software programs are now commonly known as Building Information Modeling, (BIM), Virtual Building, Parametric Modeling or Model-Based Design [43].

The latest developments in BIM technology mean that all of the 3D building objects created in the design phase can coexist in a single “project database,” or “virtual building”, that captures everything known about the building. A building information model (in theory) provides a single, logical, consistent source for all information associated with the building. Instead of representing a wall two-dimensionally with two parallel lines, the wall object has properties that describe geometrical dimensions such as length, width and height as well as materials, finishes, specifications, manufacturer, and price which are also included. Doors, windows, slabs, structural members, and stairs can be objectified in the same way [44].

Additionally, the location of an object within a built asset can be pinpointed using unique geospatial referencing [45] which can be incorporated into the model. An example of these relations is the following strain of links from an object: “A duct, having an asset code of BSE-DU694 is installed on building storey Level 3 of the building named Block B situated on a land parcel with Lot No 1222546” [45].

In BIM, the model comprises individual built assets, sites or geographic information system (i.e., precise geometric coordinates coupled with accurate geometry and represented visually), with attributes that define their detailed description and relationships, that specify the nature of the context with other objects. Because all components within a BIM are objectified and have properties and relationships attached to them, BIM is called a “rich” model. In this way, BIM offers a variety of information that is generated automatically as the design model is created. In turn, this information can be used for cost estimating, project planning and control, and sustainability (such as Life Cycle Analysis and Life Cycle Costing) and eventually for management of the operation and maintenance of the built asset [37]. 

For government, BIM offers a digital modeling technology that offers the potential to integrate design, engineering, and construction maintenance and decommissioning information about a built asset project into a single “rich” model. Further, BIM technology enables the use of 3D drawings to be moved beyond the design phase and into the construction, maintenance, and decommissioning phases of the built asset although few projects have been able to demonstrate this level of functionality to date.

#### 4.4.2. The Utility of BIM in the Construction Phase

The application of BIM to the construction phase is possible because the underlying data of the BIM contains rich data concerning not just individual elements of the model but also the relationships between these elements. Cyon Research [46] provides an example: “although a door has an independent existence, it will move with a wall in which it has been inserted.” (Design professionals will recognise that the concept of “parametric integrity” is being discussed here.) For designers and builders, this means that amendments to building designs can be made rapidly, easily, and accurately as all of the related elements of a particular drawing are adjusted at the same time. 

While the models created in BIM software are detailed 3D representations of built assets, they are more than that. Although BIM can create 3D visualization, the model is not constructed from simple graphical elements. Instead, it is generated from a relational database containing information regarding the elements of a structure and their relationships [5]. Built asset elements can contain many nongeometric attributes, fire resistance, for example, or manufacturer's name and model number. This makes for a realistic model: one whose every aspect is linked to every other aspect to reflect reality. A change made to any “view” of the model, whether graphical or textual, is immediately reflected in every other view [46].

The elements of a built asset in a BIM model can include data concerning their composition, cost, manufacturer, relationship to other elements, and related properties such as dimensions, weight, fire resistance, or combustibility. Such information becomes very useful for estimating costs and bills of quantity and so forth. 

An extra addition to this 3D parametric modeling is that it is also the basis for the possibility to apply 4D simulations. 4D is industry code for the addition of the element of time to a built asset model. A 4D simulation program is a software tool that automatically prepares construction schedules together to show a 3D simulation of the construction progress over time—which is where the idea of 4D originates. The process of assigning time to each of the elements of a 3D built asset model greatly reduces the chance of human errors in the construction process. Also the visualisation of the construction process enhances the understanding of the process involved, so that any issues can be identified by nontechnical people, and the visualisation may highlight constraints that had not been previously identified or made explicit [47]. A 4D BIM can greatly enhance traditional project management software as the specific visual representation of construction elements is linked to specific points in time. A client and builder can see a visual representation of the state of the finalised building at any given point of time. 

An example of this was given in the construction of a hospital, where the 4D model was shown to the clients (who worked for a large hospital) prior to construction [48]. The 4D model showed all the stages of the construction process, including the equipment needed to construct the built asset. When viewing the construction process, the gantries and large cranes planned to erect the building were also displayed. The hospital staff quickly pointed out that this equipment blocked the primary flight path of emergency helicopters to the helipad essential to the rest of the hospital; therefore, such equipment could not be used. This had obvious implications for the construction planning process, and considerable effort was required in order to resolve this issue. However, it was resolved prior to commencement of construction, thereby demonstrating the utility of BIM for not just the planning phase but also the construction phase. 

Williams [49] provides a useful overview of how BIM has been used in the construction of a variety of transport infrastructure including bridges, viaducts, and railway tunnels. 4D BIM is used in these applications to not only demonstrate the construction of the infrastructure itself but also to show how traffic could keep flowing, although rerouted, at different stages of a subway construction, how a section of a viaduct could be demolished and rebuilt in only 3 days, and what various construction options would look like if implemented. Examples of this are above ground versus underground highways, the impact this would have on a city's foreshore, and what the construction of a high rise building would look like at different times of the year. 

There are also advantages for subcontractors involved in the construction phase as the detailed designs facilitate computer-controlled manufacturing, automated estimated/quoting, and accurate off-site manufacture resulting in improved coordination, reduced time, and less waste. 

For government, BIM provides a way to better engage with clients in the design phase but can also result in significant productivity improvements in the delivery of the built asset. The ability to provide detailed model, which contains detailed specification of a built asset in one place, enables the rapid identification of errors and collaboration between various design professionals. The functionality goes beyond the specifics of the built asset itself and has also been applied to the access, egress, traffic, equipment, and other elements essential to the effective running of the project. 

Just as the introduction of BIM in the design phase has indirect advantages for the government, so does the introduction of BIM in the construction phase. BIM allows for better cost estimates of the project. As every phase of the construction is modeled, unforeseen costs can be eliminated in the planning phase, instead of “fire fighting” behavior during the project, which in general costs more time and money than estimated. In addition, it also leads to a better service quality delivery, increased consultation, reduced disputes, and reduced lead time. These advantages of BIM to the government will be discussed in detail later.

#### 4.4.3. The Utility of BIM in the Operations/Maintenance Phase

BIM also has applicability to the maintenance phase of built assets. Since all the specifications for a built asset, down to an individual component level, are recorded, BIM provides a repository of detailed information about the built asset and its components that can be used after the completion of the built asset for Facility Management (FM). The FM has easy and quick access to important information during the maintenance phase and moreover can update this information over time, which can result in better management of the asset. This framework also means that the owner of the built asset can easily change from one facility manager to another, as only one single BIM file needs to be exchanged [50, 51]. 

Facilities Management is “a business practice that optimises people, process, assets, and the work environment to support delivery of the organisation's business objectives” [52]. If maintained properly during construction, BIM can become a tool that can be used by the owner to manage and operate the structure or facility [5]. According to the CRC Construction Innovation [9], facilities management is one of Australia's fastest growing industries which contributes significantly to the economy and employs a great number of people. Recent statistics on the FM industry support this contention. The combined direct and indirect contribution of the FM industry in 2002/2003 was AUS $12.2 billion of value added, AUS $12.4 billion in GDP terms, and (full time equivalent) employment of 172,000 persons. The combined contributions represented 1.8%, 1.65%, and 2.1% share of the corresponding Australian GDP and employment totals [13]. 

The addition of BIM in the maintenance phase has particular utility for governments in the maintenance of a built asset. As BIM can contain all of the data concerning the components of a built asset, the condition of these components can also be entered and audited. Given the typical longevity of government built assets and the current regime of contracting-out asset management to private firms, any given asset may have multiple firms contracted to maintain them. This presents a challenge for effective asset management, as the change of firms often results in loss of local knowledge about the built asset itself. BIM provides a tool which can retain records of all the updated data of the built asset. Additionally, if a particular building element was to fail, then the constructor or supplier of that particular asset could be readily identified and contacted to provide a replacement element. 

As there is one knowledge base for a built asset, multiple firms can be used to manage the built asset, as every facility manager would update the BIM with additional information. Such a database would also provide a basis for auditing the performance of the facilities manager as well as the performance of the built asset itself. Additionally, switching from one facility manager to another is simplified, as the BIM contains all the needed information for a new facility manager. Thus, BIM has the potential to reduce opportunistic behaviour from the facility manager and creates incentives for the facility manager to perform as best as possible.

For government, there would appear to be great benefit in using BIM models for FM applications, as BIM can be used to integrate “a digital description of a built asset with all the elements that contribute to its ongoing function such as air conditioning, maintenance, cleaning, or refurbishment and describe the relationship between each element” [47]. The Sydney Opera House FM Exemplar Project is an example of BIM used as a facilities maintenance tool [13, 47, 53]. There are advantages to a computer model which can be handed on from one contractor to another in contracting-out regimes, the primary advantage being continuity of available information from one FM contractor to the next, thus enhancing the stewardship of such assets in contracting-out regimes. 

#### 4.4.4. The Utility of BIM in the Decommissioning Phase

At the end of the built asset life, when it is decommissioned, BIM is useful in supplying the information of the built asset construction, materials, and the whole life history. From the BIM, information about hazardous materials or elements used in the built asset or in repair work can be identified, and these can be extracted and stored as deemed appropriate. This readily available data will increase the speed at which the built asset can be decommissioned and will also increase the safety of the decommissioning. As some built asset products are only deemed hazardous many years after construction (e.g., Asbestos), having a detailed database available of the built asset and the composition of its components greatly assists in the management of risk. Also, BIM increases the overall sustainability of the built asset as it allows the identification of dangerous materials that require special handling and valuable materials that can be reused. It also can assist possible future needs for dismantling built assets and reusing the entire built asset or components of a built asset, instead of simply demolishing the built asset.

For government, having a detailed model of a building ready that contains the composition of all elements of the structure, enables the identification of (potentially) hazardous materials like lead and asbestos. Some construction materials are recognised as being hazardous long after their incorporation into the built asset. Just like the evolution of asbestos from a renowned built asset material to a dangerous substance, currently used materials can in the future be classified as dangerous. The availability of a BIM can help in identifying where and how often these materials were used. Additionally, some components of a built asset could still hold considerable value, such as copper, and could be reused in an economically viable manner. 

## 5. Addressing the Challenges

The challenges as set out in the previous section need to be addressed when BIM is desired to be implemented. Addressing these points will increase its strength and output. In this section some suggestions to mitigate the disadvantages are discussed. 

### 5.1. A Single Detailed Model

Even though BIM is a single detailed model, this should not limit the possibilities of experimenting with different versions in the design phase. In this phase, if desired, two or more initial models could be initiated, giving the designer room to experiment with alternative design schemes. This is assumed to take up a lot of storage space, but as the design progresses, in time only one design will remain. Together with the latest advances in IT technology, which allows for increasing storage capacity this disadvantage could become less challenging. 

### 5.2. Interoperability

As it is vital for the success of BIM that all participating parties of the project use the same programs, the same versions of programs, and IFC standards, this will have to be accomplished before starting the project. In the initiation stage, all participants will have to agree on switching to the new standard if they are not using these yet. Another option would be only entering arrangements with partners that comply with the requirements beforehand. In this way interoperability challenges are addressed, as incompatibilities leading to delays can be avoided. For example, the UK government will require fully collaborative 3D BIM as a minimum, by 2016 [2].

### 5.3. Added Work for the Designer

Certain incentives for the designers and architects of the model will have to be integrated in the contracting process, as the initial creators of the model, the designers, and architects have a big influence on the future development of the model. The initial design stage therefore carries extra responsibilities, liabilities, and work. Straightforward rewards of money can be offered to the designer to compensate them for the work, or certain arrangements like royalties for artists could be incorporated when the underlying data of the model is used again. However, these are just suggestions and have as such not yet been researched whether this is possible or legally achievable. It is however clear that the job description of the architect changes in BIM processes. Some of the issues which need to be resolved for this to happen, particularly IP, are to be discussed in more detail.

### 5.4. The Size and Complexity of BIM

As addressed earlier, developments in internet and computer technology have greatly enabled the possibilities of larger and more complex technological projects. In general, just the storage of the model should not create the largest of problems as storage space becomes increasingly cheaper. The larger difficulties will arise in creating real-time access and sharing of the database; ubiquitous high speed broadband internet is essential, together with ways of ensuring the data is secure, stable, and accessible. The key here is generating and/or accessing the right data for the right purpose, rather than accessing all the data.

## 6. Conclusions and Recommendations

This paper outlines the current potential of BIM to enhance the productivity of civil engineering. The promise and advantages of an integrated information and database sharing model across the entire life span of a built asset have been identified, together with the current barriers to implementing such models on a large scale. Of special interest to the industry are the potential cost savings BIM promises to deliver—particularly through improved efficiencies and effectiveness through enhanced collaboration at all stages of the construction cycle. Recent advances in IT (both hardware and software) have enabled advanced knowledge management, which in turn facilitates sustainability and improved asset management in the civil construction industry.

Many of the tools and technology that have been discussed in this paper are embedded in daily work practices of civil engineers already. The main challenges are not the interconnection of software tools anymore, but rather establishing processes and best practices, overcoming the barriers, and managing the social element of sociotechnical systems. [54] said that research indicates that one of the last available “mechanisms” left for organisations to improve their competitive position is by considering its people (culture) along with its technology [54]. In other words it is not the technology itself that we should be focusing on anymore, but the process of interaction between architects, engineers, constructors, and government. 

Such interactions have implications for the types of procurement arrangements which would facilitate such interactions, together with the contractual, legal, financial, and technological frameworks needed to support the implementation of BIM and the amelioration of some of the difficulties associated with implementing BIM. However, it was already noted by Williams and Dobson [55] that changing the culture of an organisation and its members takes time. That is because it is a slow process for people in existing or newly established “social systems” to develop a new set of common held beliefs, attitudes, and values [55]. What this means for BIM is that changing current ways of working will not make BIM an instant success but is a pathway to future success. Many firms adopt ICT tools and systems for profit-motivated reasons and often fail due to underestimating the social implications of the change brought by the innovation. Successful ICT adoption depends on the “politics of technology” in its management in the organisation [54].

BIM does have significant potential for civil engineering. The government, as a significant client of construction, has been called upon to be an early adopter of the technology [33] and in cases like the UK [2] and the USA [38], they have taken that role. Pilots in various countries have demonstrated significant time, costs savings, and quality enhancements. There are however significant barriers and costs which need to be addressed in order for these benefits to be realised on a broad scale, as discussed here. It is therefore recommended that several civil engineering industries maintain undertaking small and some larger projects, in order to assess the benefits of the technology and to work through the numerous issues raised in this paper. Additionally, these projects could be conducted in different jurisdictions and for different clients, as such variables are likely to provide valuable lessons which have purchase in wider contexts. 

Given the barriers identified above, a further review of the interorganisational, legal, public policy, and financial issues inhibiting the implementation of BIM is advised. 

Digital construction is coming, and the implementation of BIM is very likely to become reality, maybe very quickly or maybe at a more leisurely pace. What the civil engineering industry should do is to make sure that it is leading the world, and continue to invest in digital capabilities to continuously improve efficiencies and effectiveness through enhanced collaboration at all stages of the construction cycle.

## Figures and Tables

**Table 1:** The application of BIM to the asset life cycle (adapted and expanded from Hartman and Fischer [3]*).

Design	Construction	Operations/facilities management	Decommissioning
Ensure the right facility is designed. Evaluate the design from many perspectives. Evaluate the design against building codes and sustainability before construction.	More productive crews, as there are fewer changes to the design once the construction has started, the ability to track work in real time, faster flow of resources, and site management. Enables demonstration of the construction process, including access and egress, traffic flows, site materials, machinery, and so forth. Better tracking of cost control and cash flow, particularly for large projects.	Keep track of built asset. Manage the facility proactively. Such a model can be handed from one contractor to another, thus enhancing facilities management. Capability to schedule maintenance and review maintenance history.	Identify elements which can be recycled or those which require particular care (e.g., hazardous materials). Know the composition of structures prior to demolition.

*Hartman and Fischer [3] also argue that BIM can enhance procurement processes. However, procurement could apply to any or all of the construction phases discussed here. Likewise Building Smart (2007) argued that BIM had application for increasing the speed of Development Assessment approvals through local councils.

**Table 2:** Overview of applications of BIM in the current and past projects.

Project or organisation	Role of BIM within the project
The Sydney Opera House [9]	BIM as a support for integrated facility management
The Construction Operations Building Information Exchange (COBIE) project [10]	COBIE is creating a standardized content and format for information handover to operations and maintenance, as a part of the U.S. National BIM standard (NBIMS)
Public schools in Bourgogne (France) [12]	All the public schools of the region will progressively become available in IFC format (CADOLE project, funded by the region as facility manager)
US General Services Administration (GSA) [11]	The GSA has created its own 3D-4D BIM Program. Starting from 2007, the GSA has mandated the use of interoperability and IFC for all major projects. This followed a pilot study which tested BIM on 9 separate projects
The US Pentagon Renovation and Construction Program (PENREN) [56]	The US PENREN Program uses the diagnostic Postoccupancy Evaluation (POE) process which systematically evaluates the performance of built assets after they have been built and occupied for a length of time
The Airbus restaurant in France (IAI 2006)	Main purpose: to populate the FM system with IFC files provided by the designers
The Freedom Tower in New York City [30].	When completed, the Freedom Tower will be 1,776 feet tall, the world's tallest built asset, and contain approximately 2.6 million square feet. Given the high visibility and aggressive schedule associated with such a large, complex project, SOM's commitment to a full BIM approach to the project is both a bold bet and the only realistic way to deliver on the unique demands of this project
The UK Process Protocol model [57]	Based on a 2D model, the process protocol adopts a normative approach to inspire companies to use a more disciplined strategy to project management
The model from the Finnish Construction Process [58]	The Technical Research Centre of Finland created a model that followed existing practice quite close, since the input consisted of checklists of tasks produced by the Built asset Information Institute, which are the ‘‘de facto” standard in Finland
The IBPM of Pennsylvania State University [59]	The work carried out by the Pennsylvania State University has a large influence on the later work in formalised modelling technology. It was carried out with close collaboration with industry and real projects
The Dutch ‘‘Bouw informatie model” [60]	They used BIM in essence as a design process model, intended to serve as a framework for describing information in the creation and modelling of the model
Queensland State Archives, Runcorn	This is pilot project conducted by the Department of Public Works where a 3D model was developed from 2D drawings and used for the design and construct phase. The project is currently being evaluated. A 4D construction scheduling was also a key element of this project
US Army Corps of Engineers	The US Army Corps of Engineers and a global engineering, procurement, and construction management (EPCM) firm,as a partnership, work together to develop new US Army Corps of Engineers (USACE) project deliverable standards for BIM applications

**Table 3:** Overview of benefits of BIM to the asset life cycle stages.

	Planning	Construction	Facilities management	Decommissioning
Sydney Opera House			Faster and more effective processes. Information is more easily shared and reused/controlled whole-of-life costs and environmental data Environmental performance is more predictable and life cycle costs are understood	Identify elements which can be recycled or those which require particular care (e.g., hazardous materials)
COBIE	Creating standardized content and format for information handover to construction	Better tracking of cost control and cash flow, particularly for large projects	Creating standardized content and format for information handover to operations and maintenance	Identify elements which can be recycled or those which require particular care (e.g., hazardous materials)
GSA BIM	Evaluate the design from many perspectives and through time	Enables demonstration of the construction process, including access and egress, traffic flows, site materials, machinery, and so forth	Capability to schedule maintenance and review maintenance history	Know the composition of structures prior to demolition
US Army Corps	Evaluate the design against building codes and sustainability before construction	More productive crews, as there are fewer changes to design once the construction has started, the ability to track work in real time, faster flow of resources, and site management	Manage the facility proactively	Identify elements which can be recycled or those which require particular care (e.g., hazardous materials)

**Table 4:** The benefits of using business information modelling.

Benefit	Result
Increased utility and speed (in all phases)	Information is more easily shared, can be value-added, and reused
Enhanced collaborations (mainly in the design and construction phase)	Across discipline and organisation, built asset proposals can be rigorously analysed, simulations can be performed quickly, and performance benchmarked, enabling improved and innovative solutions
Better data quality (in all phases)	Documentation output is flexible and exploits automation. Requirements, design, construction, and operational information can be used in FM resulting in better management of assets
Visualisation of data (mainly in the design and construction phase)	The added value of 3D visualization leaves little room for misinterpretation by all parties involved, and it helps to realign their expectations
Enhanced fault finding (in all phases)	BIM greatly reduces conflict issues by integrating all the key systems into the model. Designing BIM systems can detect internal conflicts, and model viewing systems can detect and highlight conflicts between the models and other information imported into the viewer

The key advantage of BIM is its accurate geometrical representation of all the parts of a built asset capturing all necessary and relevant data of every part in an integrated environment.

**Table 5:** The disadvantages of built asset information modelling.

Disadvantages of BIM	Description
A single detailed model	BIM does not allow alternative design options or the managing of ‘‘what if” scenarios.
Interoperability	One software standard needed. Often firms have their own software; for BIM, every firm needs to change to the same software standard throughout the entire built asset process.
Added work for the designer	For BIM to work ‘‘optima forma”, the designer needs to create the ‘‘rich” model. They will be drawing something that will form the foundations of a complete system analysis. This means a lot more work for the designer.
The size and complexity of BIM	The large size of BIM will require different means of data sharing, and real time access into the database will require broadband internet access, together with security of data being worked on.
Trade Practices implications	While some countries have mandated BIM, this is unlikely to occur in Australia, if this restricted trade.

**Table 6:** The barriers to the implementation of built asset information modelling.

Barriers of implementation	Description
Issues concerning IP, liability, risks, and contracts	As the designer is responsible for the creation of the ‘‘rich” model that will be used throughout the process, this raises issues of who owns the IP, who is liable, what are the risks involved, and how will new contracts be structured?
Issues concerning the authenticity of users	Using electronic environments for tendering raises authenticity questions because manipulation of data may be possible, and the authenticity of users needs to be secured.
Costs	For designers, the economic advantages of BIM are less tangible. Yet, it is the designer, not the owner, that must adopt and invest in the new technology, So unless the designer shares in the economic benefits, the owner, not the designer, reaps the immediate benefits and the designer pays the price. Builders and owners benefit significantly from BIM.
Sociotechnical issues	Attention needs to be given to the socio-technical issues which arise from implementation of new technology, which results in new ways of doing business.
Skill issues	Access to BIM may be limited or inhibited by users either not having the capability or the ‘‘know how” in terms of connecting to the system. Obtaining sufficient level of knowledge and expertise that is required for BIM may be difficult and prohibitively expensive.
